# Sex chromosomes/hormones and the tumor microenvironment of non-reproductive cancers

**DOI:** 10.3389/fimmu.2025.1642956

**Published:** 2025-09-12

**Authors:** Chun-Miao Zhang, Zhong-Bo Ge, Hai-Hong Zhou, Meng-Xiao Wei, Xin-Yuan Ding, Zhe-Zheng Lin, Ming-Yu Wang, Cai-Juan Bai

**Affiliations:** ^1^ Key Laboratory of Preclinical Study for New Drugs of Gansu Province, Institute of Biochemistry and Molecular Biology, School of Basic Medical Sciences, Lanzhou University, Lanzhou, China; ^2^ Ministry Of Education Key Laboratory of Cell Activities and Stress Adaptations, School of Life Sciences, Lanzhou University, Lanzhou, China; ^3^ Centre for Translational Medicine, Gansu Provincial Academic Institute for Medical Research, Lanzhou, China; ^4^ Centre for Translational Medicine, Sun Yat-Sen University Cancer Center Gansu Hospital, Lanzhou, China; ^5^ National Health Commission Key Laboratory of Diagnosis and Therapy of Gastrointestinal Tumor, Gansu Provincial Hospital, Lanzhou, China; ^6^ The Institute of Clinical Research and Translational Medicine, Gansu Provincial Hospital, Lanzhou, China

**Keywords:** sexual dimorphism, tumor microenvironment, loss of Y chromosome, X-chromosome inactivation escape, sex hormone, antitumor immunity, non-reproductive cancer

## Abstract

Cancer exhibits profound sexual dimorphism in incidence and therapeutic outcomes, driven by the interplay between biological sex determinants and immune regulation. Besides established environmental risk factors (e.g., male-predominant smoking/alcohol consumption), emerging evidence identifies the tumor immune microenvironment (TIME) as a pivotal mediator of sex disparities in carcinogenesis and immunotherapy response. This review synthesizes recent advances in two fundamental mechanisms: (1) Sex chromosome biology: Recent studies delineate the Ubiquitous loss of chromosome Y (LOY) of male cancers that promotes immunosuppressive TIME remodeling, while X-chromosome inactivation escape in females enhances antitumor immunity; (2) Endocrine regulation: Androgen receptor signaling induces T-cell exhaustion via PD-1 transcriptional activation in males. Estrogen-ERα boosts cancer progression via PD-L1 high expression, whereas ERβ inhibits cancer progression via CD8^+^ T cell activation in females. This mechanistic synthesis provides actionable strategies for precision immuno-oncology trials targeting sex-based immunological divergence.

## 1 Introduction

Cancer remains a leading cause of global mortality (1). Population-based studies reveal significant sex-based disparities in the incidence of most non-reproductive cancers (2). This dichotomy extends to mortality patterns, where male predominance persists in lung, colorectal, and gastric cancers (3, 4). Accumulating evidence indicates that cancer-related sex disparities are mediated through multifactorial mechanisms encompassing lifestyle exposures, chromosomal determinants and hormonal regulation (5–7).

Lifestyle factors (dietary patterns, smoking and alcohol consumption) contribute to sex-specific cancer disparities. For instance, a prospective cohort study demonstrates that low-fat/high-fiber dietary patterns significantly reduce the risk of colorectal cancer specifically in males, suggesting biological susceptibility to diet-modulated carcinogenesis (8). Similarly, nationwide registry data identify persistent smoking disparities as drivers of elevated lung cancer mortality in Chinese males (3). In addition, multiple population-based cohort studies have demonstrated significantly higher alcohol-associated cancer mortality (including primary liver cancer, colorectal cancer, and esophageal cancer) in males compared to females (9, 10). This disparity primarily stems from the higher prevalence of risk-lifestyle among male populations. However, following rigorous adjustment for lifestyle confounders, epidemiological analyses consistently demonstrate persistently elevated incidence and mortality ratios of multiple malignancies in males, which are potentially mediated by sex-specific chromosome determinants or hormonal regulation (2, 11–13).

Sex chromosome complement constitutes key determinants of sex-based cancer disparities (12–14) through multilayered regulatory mechanisms. Notably, A subset of X-chromosome genes can escape X-inactivation, which would protect females from complete functional loss and confer relative tolerance to carcinogenesis (14). Conversely, males exhibit X-monosomy vulnerability, so X-linked tumor suppressor loss-of-function mutations would directly drive carcinogenesis. Beyond these cell-autonomous effects, emerging evidence highlights the pivotal regulatory roles of immune microenvironment remodeling in oncogenesis and its progression (15–17). Notably, the sex chromosome harbors a large number of immune-related genes and exerts cancer-modulating effects through spatiotemporal reprogramming of tumor-immune interfaces (18). Concurrently, sex steroids, including estrogens and androgens, have profound effects on immune function which could affect autoimmunity, allergy, infectious diseases, and cancers (19).

The tumor immune microenvironment exhibits sex-specific remodeling through chromosomal dosage effects (XX vs. XaY) and steroid hormone signaling gradients. These molecular mechanisms partially explain the observed sexual dimorphism in cancer incidence and treatment outcomes. Research based on the Four Core Genotypes (FCG) model (20, 21) reveals that sex chromosomes and sex hormones often coordinate or compensate for regulation (22). Notwithstanding these complexities, this review systematizes current findings on the regulatory dynamics of sex chromosomes and sex hormones within the tumor immune microenvironment. This review underscores the imperative to recognize sexual dimorphism in cancer pathogenesis, advocating for the integration of sex-stratified precision therapeutic frameworks to optimize clinical outcomes through personalized intervention paradigms.

## 2 Sex chromosome-mediated sexual dimorphism in cancer

The X and Y chromosomes constitute distinct tumor microenvironments, with Y-chromosome loss events and X-chromosome inactivation (XCI) escape mechanisms.

The emergence of sexual dimorphism in cancer incidence and outcomes is mechanistically driven by sex-biased reconfiguration of the tumor microenvironment, particularly involving dysregulation of T cell exhaustion and cancer/testis antigens (CTAs). Deciphering the interplay between sex chromosome dynamics (LOY, XCI escape) and immunotherapy responsiveness will advance the development of sex-specific biomarkers and targeted therapeutic strategies.

### 2.1 Y chromosome

**Figure 1:**
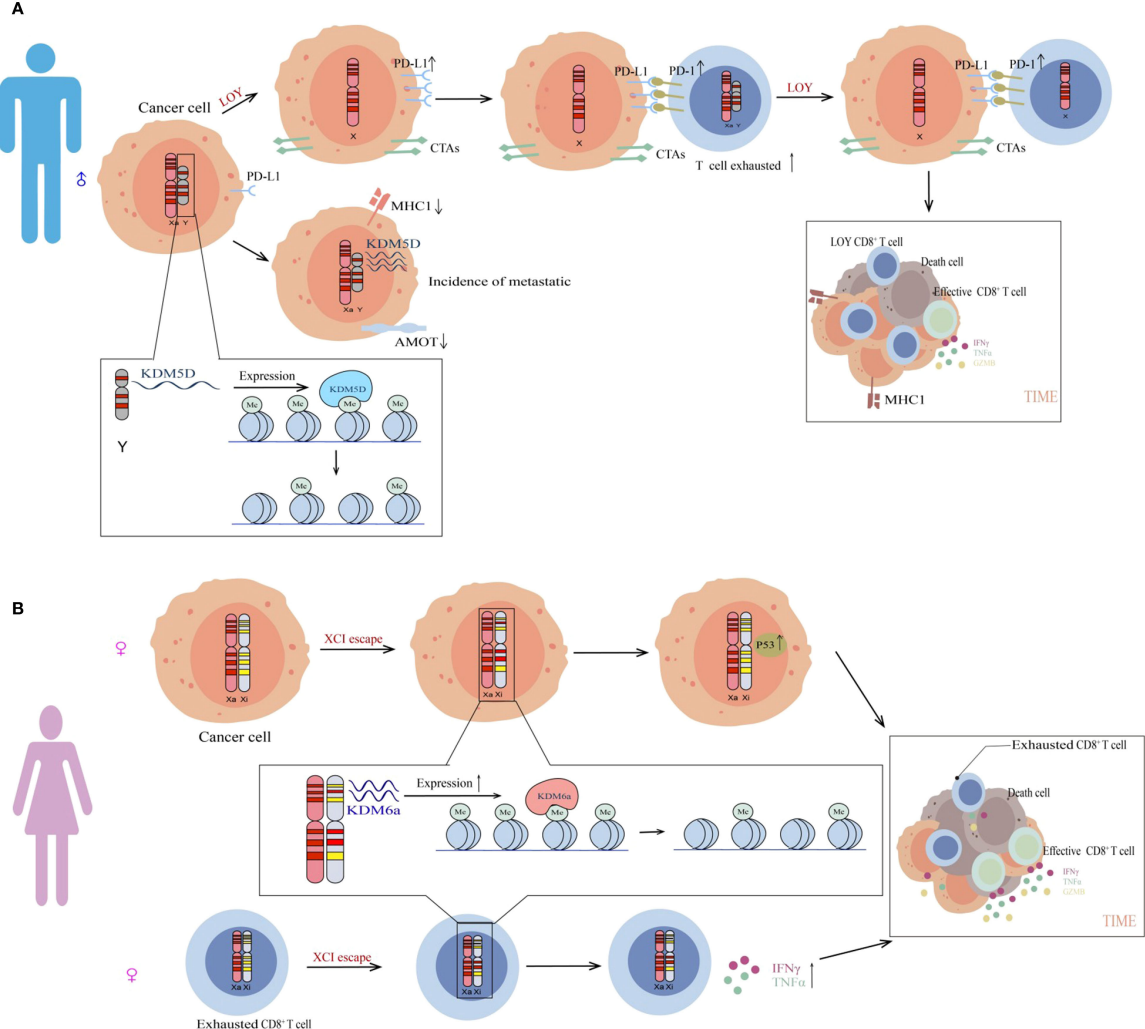
The role of chromosomes in cancer sexual dimorphism. **(A)** Loss of the Y chromosome in tumor cells from male patients contributes to the tumor immunosuppressive microenvironment. Compared to Y chromosome-retained tumors, LOY tumors exhibit heightened immunosuppression and T cell exhaustion. Meanwhile, tumor cells lacking the Y chromosome may induce LOY in T cells and recruit pre-existing LOY T cells, fostering a suppressive immune microenvironment. Furthermore, the *KDM5D* gene epigenetically suppresses *MHC-I* and *AMOT* expression, facilitating tumor immune evasion. **(B)** X-chromosome inactivation escape promotes antitumor immunity in females. XCI escape enables biallelic expression of genes like *KDM6A* in females (XX). Elevated Kdm6a protein activates the p53 pathway in tumor cells, suppressing tumor growth. Additionally, *KDM6A* restores cytotoxic activity in exhausted T cells, further inhibiting tumor progression.

LOY represents a common somatic alteration in male cancer patients, frequently correlating with poor prognosis in elderly males (23–26). Emerging research reveals substantial overlap between LOY-associated genomic variants and known cancer susceptibility loci, somatic drivers of tumor progression, and genes targeted by approved or investigational anticancer therapies (27). The analysis of The Cancer Genome Atlas Program (TCGA) database cohort suggests that LOY is present in early-stage cancers and serves as a poor prognostic indicator for various tumors in men (28). In a pivotal study investigating the association of LOY with adverse outcomes in bladder cancer, TCGA data analysis demonstrated that patients with reduced expression of Y chromosome-encoded genes (e.g., *KDM5D* and *UTY also known as KDM6C*) exhibited shorter survival, with LOY detectable even in early-stage malignancies (29). LOY drives tumor immunosuppression by altering secretory factors or surface molecules in cancer cells, upregulating T cell exhaustion markers (e.g., *TOX*, *TIM-3*, *LAG-3*), and recruiting M2-polarized macrophages to form an immunosuppressive microenvironment (**Figure 1A**). The latest published results reconfirmed these conclusions through the “Y chromosome transcription signature” (YchrS) in clinical samples (30). Beyond the LOY observed in neoplastic cells, significant prevalence was detected in tumor-infiltrating immune cells, particularly within T-cell populations. Further analysis revealed a direct correlation between immune cell LOY and T-cell-mediated immunosuppression (30). Notably, Y-deficient tumor models displayed enhanced responsiveness to PD-1 inhibitors, with post-treatment reactivation of CD8^+^ T cells from an exhausted to an activated state. Clinical data corroborate that LOY-positive patients achieve significantly improved survival following anti-PD-1 therapy (28, 29).

*KDM5D*, encoded by the Y chromosome, is a histone-modifying enzyme (31). Obviously, LOY will inevitably lead to abnormal function of the *KDM5D* gene. Jiexi Li et al. recently reported that Y-chromosomal *KDM5D* drives male bias in *KRAS*-mutant colorectal cancer (31). *KRAS* mutation downregulates *KDM5D* expression through *STAT4* inactivation, which enhances tumor invasiveness and metastatic potential via dysfunctional CD8^+^ T cells. Murine models showed that *KDM5D* deletion reduced tumor aggressiveness and augmented CD8^+^ T cell-mediated cytotoxicity (31). Mechanistically, *KDM5D* epigenetically suppresses *AMOT*, a gene critical for maintaining intercellular tight junctions, thereby promoting metastasis. Notably, *KDM5D* also diminishes MHC class I antigen presentation and CD8^+^ T cell-mediated cytotoxicity, enabling immune evasion (**Figure 1A**) (31).

Studies in hematologic malignancies further implicate LOY in male hematopoietic cells as a critical driver of leukemogenesis and disease progression (32, 33). Intriguingly, male patients receiving female-derived hematopoietic stem cell transplants exhibit elevated relapse risk, potentially attributable to weakened graft-versus-leukemia effects due to sex-mismatched H-Y antigen expression encoded by the Y chromosome (34).

Recent findings by Jonas Fischer et al. demonstrate that LOY in lung adenocarcinoma remodels tumor immunogenicity via dysregulation of CTAs, facilitating immune evasion and significantly impacting survival outcomes in pembrolizumab-treated cohorts (35). Collectively, these discoveries suggest LOY quantification may serve as a biomarker for personalized immunotherapy selection, while CTA-targeted immunotherapies could synergize with existing regimens to enhance therapeutic efficacy.

### 2.2 X chromosome

XCI is an epigenetic mechanism that silences one X chromosome in female cells to balance X-linked gene expression between XX and XY individuals (36, 37). However, approximately 15–25% of X-chromosomal genes escape XCI (termed “escape genes”) (38), many of which exhibit higher expression levels in females than males, particularly those involved in immunity and tumor suppression, such as Toll-like receptor 7 (*TLR7*) and *KDM6A* (39, 40). Biallelic expression of these escape genes enhances antitumor functionality in female immune cells, contributing to lower incidence and mortality rates in females for cancers like bladder cancer.

For the past few years, researchers have increasingly focused on the *KDM6A* gene*. KDM6A*, an X-chromosome inactivation escape gene, exhibits significantly higher expression in female cells compared to males (41). Although both *KDM6A* and *KDM5D* belong to the lysine demethylase superfamily, they exhibit substrate specificity for distinct histone lysine residues—*KDM6A* catalyzing H3K27me3 demethylation and *KDM5D* targeting H3K4me3 (42). Previous studies have shown that *KDM6A* contributes to sex disparities in bladder cancer (BCa) with 3–5 times more protective effects in females (39). This study demonstrates that female bladder epithelial cells with elevated *KDM6A* expression activate p53 downstream targets (e.g., *Cdkn1a*, *Perp*) through removal of the transcriptional repressive H3K27me3, thereby inducing cell cycle arrest and apoptosis (**Figure 1B**) (39). Notably, even catalytically inactive *KDM6A* mutants partially suppress tumor cell proliferation. *KDM6A* knockout in mice significantly increased bladder cancer risk in females, while males remained unaffected due to compensation by the Y-chromosomal homolog *Uty* (homologous gene of *KDM6A* with redundant function) (39). The study demonstrates that *KDM6A* mutations or low expression correlate significantly with poor prognosis in female patients but not males. Similar sex-biased expression patterns are observed in other malignancies like clear cell renal carcinoma, suggesting tissue-specific tumor suppressor functions of *KDM6A* (39).

Similarly, a study investigating sex disparities in glioblastoma (GBM) reveals higher incidence and mortality rates in male dependent on *KDM6A* expression in CD8^+^ T cells (43). In detail, immunocompetent male mice exhibited reduced CD8^+^ T cell infiltration and enhanced exhaustion in tumor microenvironments. Male CD8^+^ T cells displayed elevated inhibitory receptor expression (*PD-1, CTLA4, LAG3*) and reduced cytokine production, whereas female tumors showed greater infiltration of effector T cells (Tef) (43). These findings suggest male T cells are more prone to exhaustion, while female T cells maintain effector functionality (**Figure 1B**). Anti-PD-1 therapy significantly prolonged survival in male mice but showed weaker efficacy in females. Meanwhile, under anti-PD-1 therapy, male tumors exhibited enhanced CD8^+^ T cell proliferative capacity and reduced exhausted T cell subsets, with minimal changes observed in females. This implies anti-PD-1 therapy primarily activates male T cell effector functions. Further analysis revealed that lower *KDM6A* levels in male T cells promote exhaustion, whereas elevated *KDM6A* expression in female T cells helps sustain effector functionality. Low expression of *KDM6A* leads to accelerated tumor growth in males with potentially greater therapeutic benefit from anti-PD-1 treatment, while maintaining superior functional capacity to constrain tumor progression in females (43).

## 3 Sex hormone-mediated immunomodulation

Sex steroids (androgens/estrogens) coordinate with sex chromosomes to establish a sex-dimorphic immune microenvironment and evoke sex-based disparities in cancer incidence and therapeutic outcomes (44).

### 3.1 Androgens

Androgen (e.g., testosterone, dihydrotestosterone) primarily exerts its effects via the androgen receptor (AR), a ligand-dependent transcription factor regulating target gene expression (45). AR is functionally active across immune cell populations, including T cells, B cells, macrophages, and neutrophils (46–48). Androgens promote T cell exhaustion in tumor-infiltrating CD8^+^ T cells by suppressing effector molecule production via AR signaling, exacerbating male-biased progression in malignancies such as bladder, colorectal, hepatocellular, and cutaneous cancers (45, 48–52).

**Figure 2:**
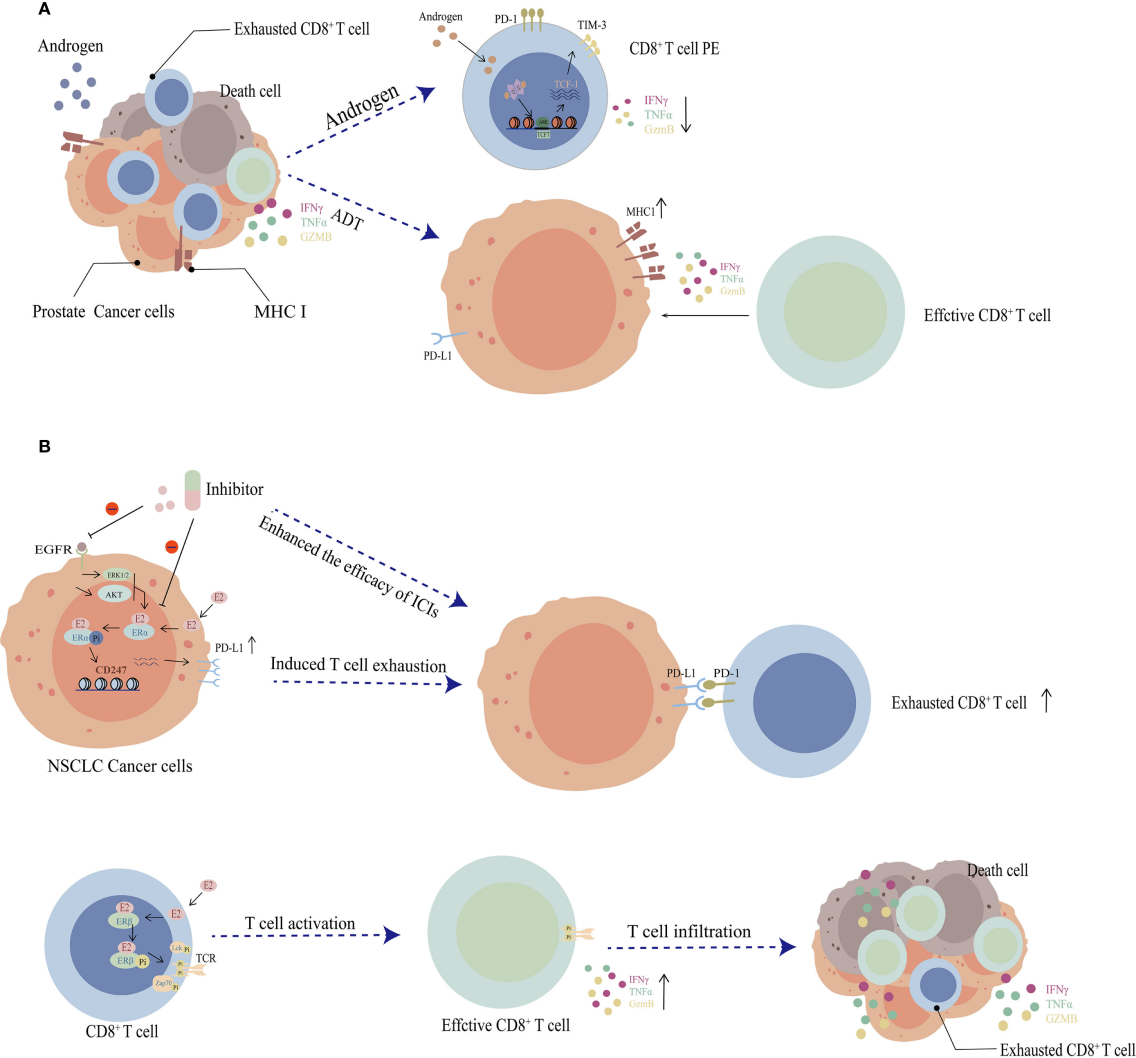
Roles of sex hormones in cancer sexual dimorphism. **(A)** Androgen receptor (AR) modulates the differentiation of tumor-infiltrating CD8^+^ T cells and impairs effector functions. AR binds to androgen response elements (AREs) located at the *TCF7* promoter, inducing its upregulation in CD8^+^ T cells. Elevated TCF1 expression promotes T cell exhaustion, thereby impairing antitumor immunity. Conversely, after androgen deprivation therapy (ADT), lower AR activity facilitates CD8^+^ T cell progression toward functional effector (Tef), enhancing antitumor immunity. These exhausted CD8^+^ T cells exhibit suppressed expression of IFNγ, TNFα and GZMB. **(B)** Estrogen activates anti-tumor immunity by binding to its receptors. E2 binding to Erα can upregulate the *CD274* expression (encoding PD-L1), driving CD8^+^ T cell exhaustion. Conversely, ERβ enhances the TCR signaling pathway (such as Zap70/Lck phosphorylation) in CD8^+^ T cells, promoting T cell activation and anti-tumor cytokine secretion (e.g., IFNγ, granzyme B, and TNFα).

Multiple studies highlight synergistic antitumor effects when combining androgen deprivation therapy (ADT) with PD-1/PD-L1 blockade (45, 48). The androgen receptor (AR) signaling pathway can directly promote the differentiation of CD8^+^ T cells into a terminally exhausted state. Mechanistically, as a transcription factor, AR specifically binds to androgen response elements (AREs) within the promoter region of the *Tcf7* gene, thereby activating its transcription (49). This leads to the upregulation of TCF-1 (encoded by *Tcf7 gene*) protein expression, which drives the progression of CD8^+^ T cell terminal exhaustion (**Figure 2A**) (49). Xiaomin Zhang et al. recently demonstrated less infiltration of CD8^+^T cells and increased expression of exhaustion markers (such as *PD-1/CD39/TIM3/TIGIT*) under ADT treatment in male mouse tumors. And castration delays tumor growth and restores T cell activity (**Figure 2A**) (53). Their work revealed that androgen signaling suppressed antitumor T cell activity through upregulating USP18, which inhibits TAK1 phosphorylation and subsequent NF-κB activation in T cells (53). In addition, Liang Chi et al. identified elevated dendritic cell (DC) subsets (cDC1, LC, cDC2) in female skin compared to males. These DCs underpin antigen presentation and adaptive immune priming and are maintained by the skin group 2 innate lymphoid cells (ILC2s). Androgens negatively regulate skin ILC2s, creating DC disparities that result in weaker adaptive antitumor immunity in males (54). Consistently, AR knockout or surgical castration will enhance antitumor T cell activity and augment PD-1 blockade efficacy in males (54).

Emerging evidence reveals that androgen receptor (AR) facilitates immune evasion in prostate cancer through transcriptional suppression of MHC class I molecules (55). Mechanistically, AR directly binds AREs within promoter regions of MHC I-associated genes (e.g., *HLA-A*, *B2M*, *TAP1/2*), inhibiting their transcriptional activity and consequently diminishing tumor antigen presentation capacity (55). AR-suppressed tumor cells exhibit heightened vulnerability to antigen-specific CD8^+^ T cell cytotoxicity in an MHC I-dependent manner (55). Notably, AR-knockdown TrampC1 tumors (TrampC1 AR-KD) demonstrated significantly restricted growth in murine models, accompanied by enhanced intra-tumoral CD8^+^ T cell infiltration and effector function (e.g., IFNγ production). However, this therapeutic effect attenuated over time, correlating with compensatory glucocorticoid receptor (GR) activation (55).

### 3.2 Estrogens

Estrogen, including estrone (E1), 17β-estradiol (E2), and estriol (E3), mainly binds estrogen receptors (ERα, ERβ, GPER1) to exert immunomodulation (56). Canonical estrogen receptor (ERα, ERβ), functioning as the intracellular receptor for estrogen, undergoes nuclear translocation upon ligand binding and specifically binds to conserved estrogen response elements within target gene promoters, thereby regulating transcriptional activation through recruitment of coactivators and chromatin remodeling complexes. Beyond canonical ER signaling, estrogens also engage membrane-bound G protein-coupled estrogen receptor 1 (GPER1) to trigger rapid response via cyclic AMP pathways (57, 58).

ERα and ERβ are expressed in various immune cells (59), but studies have shown that ERα and ERβ play distinct roles in immune cells (60, 61). The estrogen-ERα signal activates the JAK2/STAT3 pathway, driving the differentiation of bone marrow myeloid precursors into Myeloid-Derived Suppressor Cells, enhancing their immunosuppressive function, thereby inhibiting the anti-tumor T-cell response and accelerating cancer progression (62). Similarly, in female melanoma, the estrogen-ERα signal drives macrophages to polarize towards the M2 phenotype, inhibits the function of CD8^+^ T cells, promotes melanoma progression and induces immune checkpoint blockade (ICB) resistance, while the antagonist of ER (Fulvestrant) can reverse the immunosuppressive microenvironment and restore T cell function (63).

Coincidentally, in non-small cell lung cancer (NSCLC) the downstream kinases of EGFR, such as Akt, ERK1/2, will phosphorylate ERα that binds to estradiol at the Ser118 site, thereby enhancing its transcriptional activity and upregulating the expression of PD-L1 (**Figure 2B**) (64). Pharmacological intervention with the estrogen synthesis inhibitor letrozole effectively suppresses PD-L1 expression and activates CD8^+^ T/NK cells, mimicking the therapeutic effects of PD-1/PD-L1 blockade agents. Furthermore, combinatorial administration of letrozole with PD-1/PD-L1 blockade agents demonstrates synergistic efficacy, offering a promising strategy for optimizing immunotherapy outcomes, especially in 17-β-estradiol/ERα high female NSCLC patients (64).

The estrogen-ERβ pathway plays an opposite role in anti-tumor immunity. For instance, in triple-negative breast cancer and melanoma models, ERβ enhances the TCR signaling pathway (such as *Zap70/Lck* phosphorylation) in CD8^+^ T cells through the tyrosine phosphorylation switch, promoting T cell activation and anti-tumor cytokine secretion in a non-genomic manner (65). The ERβ selective agonist (S-equol) can activate this phosphorylation switch and significantly enhance the efficacy of anti-PD-1 immunotherapy, providing a new strategy to overcome ICB resistance (65). In a recently published study on gender differences in colon cancer, researchers knocked out ERβ in the intestines of female mice, which led to decreased T cell activation and infiltration in the tumor model, increased pro-inflammatory signals (*IL-6, CCL2/4*), and increased infiltration of M2-type macrophages. Additionally, TCGA cohort analysis indicated that patients with high ERβ expression had a higher survival rate (66). These results suggest that targeting and activating the estrogen-ERβ pathway can enhance the anti-tumor immune response in females.

Unlike androgens broadly suppress antitumor immunity, estrogens exhibit bidirectional (pro-/anti-inflammatory) effects depending on receptor subtypes and cellular contexts. Targeting the sex hormone-immune axis may yield sex-specific therapeutic strategies, necessitating a deeper exploration of hormone signaling dynamics and tumor microenvironment interactions (44).

## 4 Conclusions and perspectives

Males demonstrate significantly higher incidence rates and poorer prognosis across non-reproductive malignancies, with multifactorial determinants spanning sex chromosomes, sex hormones and sex-specific immune modulation (**Table 1**). Males experience LOY (27–34) and high androgen expression (53, 55), leading to an immunosuppressive microenvironment (characterized by T cell exhaustion, M2 macrophage infiltration, and downregulation of MHC-I expression) that promotes progression of multiple non-reproductive cancers. Conversely, females benefit from the biallelic expression of X-chromosome escape genes (e.g., *KDM6A*) and the bidirectional immunomodulatory effects of estrogen (39, 43). ERα signaling promotes immunosuppression (e.g., M2 macrophage polarization) (62–64), while ERβ enhances CD8^+^ T cell function (65, 66). These collectively enhance female immune surveillance capabilities, foster an anti-tumor microenvironment, and reduce cancer incidence and mortality. In general, sex chromosomes and sex hormones coordinately reshape sex-specific tumor microenvironment, and further foster sexual dimorphism in incidence and therapeutic outcomes in non-reproductive cancers.

**Table 1:** Summary of Major Risk Factors for Non-Reproductive Cancers*.

Non-reproductive cancer types	Sex bias		Related risk factors	Refs.
	Incidence rate escalation	Mortality rate escalation		
Lung cancer	Males	Males	Sex chromosomes: LOY	(27, 35, 67)
			Sex hormones: Estrogen	(63)
Colorectal Cancer	Males	Males	Sex chromosomes: *KDM5D* expression within Y	(31)
			Sex hormones: Androgen and Estrogen	(50, 51, 65)
Bladder Cancer	Males	Males	Sex chromosomes: LOY and XCI	(29, 39, 42)
			Sex hormones: Androgen	(49, 68)
Glioblastoma	Males	Males	Sex chromosomes: XCI	(39)
Skin cancer	Males	—	Sex hormones: Androgen	(47, 53)
Leukemia	Males	—	Sex chromosomes: LOY	(31)
Thyroid Cancer	Females	—	Overdiagnosis	(69, 70)
Xp11 translocation renal cell carcinoma	Females	—	Sex chromosomes: X chromosome translocation	(71)
Alveolar soft part sarcoma/FOXR2-activated central nervous system neuroblastoma	Females	—	Sex chromosomes: X chromosome translocation	(72–74)
Melanoma	Females (Premenopausal)	—	Sex hormones: Estrogen	(62, 64, 75)

*The risk factors were strictly confined to the predefined biological variables: sex chromosomes and hormones.

Studies have shown that sex differences not only lead to differences in cancer incidence rates but are also a key factor in the response to immune checkpoint inhibition therapy (13, 55). Despite the higher incidence and mortality of solid tumors in males, clinical trial data indicate superior responses to ICB in males. Meta-analyses of randomized controlled trials (RCTs) consistently report lower mortality risk in males post-ICB, though statistical significance varies across studies (76–78). In melanoma and NSCLC cohorts, males demonstrate improved overall survival (OS) and progression-free survival (PFS) following anti-PD1, anti-PDL1, or anti-CTLA4 therapy (67, 69). The observed discrepancy primarily stems from the elevated prevalence of terminally exhausted CD8^+^ T cell subsets in male patients compared to females, with these exhausted T cell populations demonstrating heightened responsiveness to ICB treatment (49, 52).

Notably, paradoxical epidemiological patterns reveal female predominance in specific non-reproductive cancer types, particularly in thyroid cancer (2, 71), Xp11 translocation renal cell carcinoma (tRCC) (75) and melanoma in pre-menopausal women (70). A high prevalence of thyroid cancer in females has been reported mainly attributed to healthcare utilization and overdiagnosis (72). The tRCC exhibits a higher incidence in females attributed to the vulnerability of the TFE3 gene translocation in the X chromosome (75). Similar mechanisms involving X-chromosome alterations are implicated in the female predominance of alveolar soft part sarcoma (ASPS) and FOXR2-activated central nervous system neuroblastoma (68, 73, 74). Melanoma predominance in pre-menopausal women is often attributed to the high estrogen levels upregulating Erα and gastrin-releasing peptide receptor (GRPR) signal (70). Therefore, female-predominant malignancies (e.g., thyroid carcinoma, tRCC) represent distinct epidemiological exceptions, while male-biased cancer incidence remains the predominant global pattern in non-reproductive cancer types.

Sex-based disparities in oncogenesis extend beyond the sex chromosome and sex hormone-mediated microenvironmental remodeling discussed herein. Many other factors could influence Sex-based disparities. Emerging data suggest that sex-associated variations in the gut microbiome directly influence innate immune responses between the sexes (79). This result demonstrates that predominant male bladder cancer patients exhibit senescence-associated neutrophil (RLSN) through defective gut microbiota-derived *Alistipes shahii* compared to females (79). In addition, pharmacokinetic sex differences, attributed to lower body weight, higher adiposity, and differential tissue perfusion in females, result in elevated drug exposure and prolonged elimination in females. For instance, ICB agents exhibit sex-dimorphic clearance. Males show faster clearance of anti-CTLA4 (tremelimumab) and anti-PD1 (nivolumab), while females metabolize anti-PDL1 (durvalumab) more rapidly (8, 80). Moreover, Age represents a significant factor in the study of sex differences in cancer. Evidence indicates that childhood tumor incidence also exhibits similar sex disparities (81). Beyond sex biology, social gender also has a multi-dimensional and throughout impact on cancer (82). A recent review article reveals that gender-sex interactions (GSI) could affect cancer biology and clinical treatment such as the timing of diagnoses, clinical trial enrolment, and the completeness of efficacy and toxicity data (82). In summary, understanding the diverse factors and mechanisms underlying sex disparities in cancer will enable optimal treatment in future clinical trials. This knowledge is crucial for developing sex-specific biomarkers (e.g., LOY, *KDM6A* and estrogen) and combination strategies targeting immune pathways.

Ultimately, research into the role of sex differences in cancer immunology holds direct translational significance. Future clinical trials should therefore be designed to maximize therapeutic efficacy and develop targeted strategies. Additionally, further investigation into whether sex-related factors can serve as biomarkers for cancer diagnosis and risk stratification will significantly enhance precision diagnostics, patient stratification, and treatment optimization.
